# Insights into the availability and distribution of oral artemisinin monotherapy in Myanmar: evidence from a nationally representative outlet survey

**DOI:** 10.1186/s12936-017-1793-0

**Published:** 2017-04-25

**Authors:** ACTwatch Group, Louis Akulayi, Angela Alum, Andrew Andrada, Julie Archer, Ekundayo D. Arogundade, Erick Auko, Abdul R. Badru, Katie Bates, Paul Bouanchaud, Meghan Bruce, Katia Bruxvoort, Peter Buyungo, Angela Camilleri, Emily D. Carter, Steven Chapman, Nikki Charman, Desmond Chavasse, Robyn Cyr, Kevin Duff, Gylsain Guedegbe, Keith Esch, Illah Evance, Anna Fulton, Hellen Gataaka, Tarryn Haslam, Emily Harris, Christine Hong, Catharine Hurley, Whitney Isenhower, Enid Kaabunga, Baraka D. Kaaya, Esther Kabui, Beth Kangwana, Lason Kapata, Henry Kaula, Gloria Kigo, Irene Kyomuhangi, Aliza Lailari, Sandra LeFevre, Megan Littrell, Greta Martin, Daniel Michael, Erik Monroe, Godefroid Mpanya, Felton Mpasela, Felix Mulama, Anne Musuva, Julius Ngigi, Edward Ngoma, Marjorie Norman, Bernard Nyauchi, Kathryn A. O’Connell, Carolyne Ochieng, Edna Ogada, Linda Ongwenyi, Ricki Orford, Saysana Phanalasy, Stephen Poyer, Justin Rahariniaina, Jacky Raharinjatovo, Lanto Razafindralambo, Solofo Razakamiadana, Christina Riley, John Rodgers, Andria Rusk, Tanya Shewchuk, Simon Sensalire, Julianna Smith, Phok Sochea, Tsione Solomon, Raymond Sudoi, Martine Esther Tassiba, Katherine Thanel, Rachel Thompson, Mitsuru Toda, Chinazo Ujuju, Marie-Alix Valensi, Vamsi Vasireddy, Cynthia B. Whitman, Cyprien Zinsou, Si Thu Thein, Hnin Su Su Khin, Aung Thi

**Affiliations:** 1 Population Services International, 1120 19th St NW Suite 600, Washington, DC 20036 USA; 2 Population Services International Myanmar, No. 16, West Shwe Gone Dine 4th Street, Yangon, Myanmar; 3 National Malaria Control Programme, Department of Public Health, Ministry of Health and Sports, Nay Pyi Taw, Myanmar

## Abstract

**Background:**

The containment of artemisinin resistance in Myanmar, historically an important probable origin and route of anti-malarial resistance to the India sub-continent and beyond, is crucial to global malaria control and elimination. This paper describes what is currently known about the sale and distribution of oral artemisinin monotherapy (AMT) across Myanmar, where this medicine is commonly found.

**Methods:**

A nationally representative 2015 outlet survey was conducted in the private sector, and among community health workers across four geographical domains. A national sample of outlets was screened for availability of malaria testing and treatment, and an audit was completed for all anti-malarials.

**Results:**

A total of 3859 outlets across Myanmar had an anti-malarial in stock on the day of survey. Of the 3859 anti-malarial stocking outlets, 988 outlets stocked oral AMT. Availability of oral AMT was highest among outlets in the Western border (36.8%) versus other domains (Eastern, 15.0%; Central, 19.3% Coastal, 10.7%). Over 90% of the oral AMT service delivery points were private sector outlets: general retailers (49.4%), pharmacies (23.5%), and itinerant drug vendors (14.2%). Eleven unique oral AMT products were audited. The most common product audited was Artesunate^®^, manufactured by Mediplantex in Vietnam, which accounted for 79.9% of the oral AMT market share. Other oral AMT products were manufactured in China and in Myanmar. Over 60% of oral AMT products had a shelf life at purchase of greater than 2 years and only 14.7% were expired. The median number of oral AMT tablets typically dispensed to treat malaria was two tablets, approximately one tenth of a full adult course. The median price of a 50 mg tablet was $0.16.

**Conclusions:**

Given the high availability and distribution of oral AMT, it is possible that Myanmar has become the last remaining viable market for any oral AMT in the region for manufacturers. National and international organizations need to act quickly and effectively to stop the production and distribution to both improve malaria control within Myanmar and reduce risk of artemisinin resistance spreading to India and Africa.

**Electronic supplementary material:**

The online version of this article (doi:10.1186/s12936-017-1793-0) contains supplementary material, which is available to authorized users.

## Background

While the World Health Organization (WHO) recommends the use of intravenous and intramuscular artemisinins for treatment of severe malaria [1], the use of oral artemisinin-based monotherapies (AMT) is not recommended for any form of malaria treatment. Oral AMT is considered to be a major contributing factor to the development of malaria parasite resistance to artemisinin derivatives [2]. Since 2007, the WHO has urged regulatory authorities in malaria-endemic countries to take measures to halt the production and marketing of these oral monotherapies, and promote access to quality-assured artemisinin-based combination therapies [3]. While many countries have adopted and enforced this resolution, the global manufacturing and production of oral AMT continues [4]. In fact, recent market intelligence data, generated by the ACTwatch project [5, 6], illustrates that oral AMT continues to be distributed and sold in some countries, particularly in the private sector. Findings from the project point to the persistent widespread availability and distribution of oral AMT in Myanmar, which accounts for the highest burden of malaria in the Greater Mekong Sub-region (GMS) and is notably considered an important probable origin and route of multi-drug resistance.

Oral artemisinin monotherapy was banned in Myanmar in 2012 by the Myanmar Food and Drug Administration (FDA). However, the regulatory policy permits oral AMT distributors with a valid 5-year import registration license awarded prior to the ban to continue to procure and sell this monotherapy within the country [7]. Given that oral AMT has a shelf life of 4 years from the time of manufacture, oral AMT medicines can be produced and imported until 2017 and could remain on the shelf until 2021. Indeed, outlet survey trend data from Eastern Myanmar over the years have shown that despite a plethora of strategies to remove this from the market, oral artemisinin monotherapy commonly persists [7]. This has included the Artemisinin Monotherapy Replacement (AMTR) project, which was designed to rapidly remove artemisinin monotherapies from the market by increasing private sector access to first-line subsidized artemisinin-based combination therapy (ACT) medicines. Additional project activities targeted private sector providers (general retailers, pharmacies and itinerant drug vendors) located in Eastern Myanmar to encourage adoption of ACT.

In 2015, the ACTwatch project for the first time, implemented a national level outlet survey in Myanmar across four geographically diverse domains to provide a complete picture of the availability and distribution of anti-malarials in the private sector and among community health workers in the not-for profit sector. The objective of this paper is to provide timely, actionable evidence to inform urgently needed strategies to remove oral AMT from Myanmar’s marketplace. This paper describes what is currently known about the sale and distribution of oral artemisinin monotherapy and answers several questions, including (1) Where is artemisinin monotherapy available and distributed? (2) What types of products are available and distributed? (3) What are the provider practices regarding the re-supply and sale of oral artemisinin monotherapy? Evidence from the oral AMT market will point to recommendations for removing this medicine completely from the market in order to protect the efficacy of artemisinins in Myanmar and beyond.

## Methods

All outlets with the potential to sell or distribute anti-malarials and/or provide malaria blood testing were included in the sample. In Myanmar, these included community health workers, private for-profit health facilities, pharmacies, general retail outlets, and itinerant drug vendors (see Additional file 1). The study was not able to gain access to government health facilities, namely public health facilities, and thus were excluded from the study.

The survey was conducted across four study domains: Eastern (where the AMTR project was implemented), Central, Western and Coastal areas of the country. A representative sample of clusters (defined as wards in urban areas and village tracts in rural areas) were selected in each domain, using probability proportional to population size (PPS). Between 180 and 240 urban wards and rural tracts in total were selected per domain. Within each selected ward/village tract, a census of all outlets with the potential to sell or distribute anti-malarials and/or provide malaria blood testing was conducted. Outlets were eligible for a provider interview and a malaria product audit if they met at least one of three study criteria: (1) one or more anti-malarials reportedly in stock the day of the survey; (2) one or more anti-malarials reportedly in stock within the 3 months preceding the survey; and/or (3) provides malaria blood testing (microscopy or rapid diagnostic test [RDT]) at the time of the survey.

Outlets that met the screening criteria were invited to participate in the survey, and among providers that gave verbal informed consent, an audit of all available anti-malarial medicines and RDT was conducted. Anti-malarial audit information included formulation, package size, brand name, active ingredients and strengths, manufacturer, country of manufacture, reported sale/distribution in the week preceding the survey, retail price, and wholesale price. Where oral artemisinin monotherapy was found, an additional module was administered to understand the supply chain for this monotherapy. Questions included where the medicine was purchased, when the medicine was last purchased from the supplier, how many packages were purchased, wholesale price, the number of tablets typically dispensed, how many packages were currently in stock, and expiry dates for packages in stock. For all interviews a structured, paper-based questionnaire was administered. Data collection was implemented from August 18th, 2015 through January 4th, 2016. Interviewer training consisted of standardized classroom presentations and exercises as well as a field exercise. Exams administered during training were used to select data collectors, supervisors, and quality-controllers. Additional training was provided for supervisors and quality-controllers focused on field monitoring, verification visits, and census procedures.

### Analysis

All data cleaning and analysis was completed using Stata 13.1 (©StataCorp, College Station, TX). Sampling weights were applied to account for variations in probability of selection, and standard error estimation accounted for clustering at the ward/village track level.

Standard ACTwatch indicator definitions have been reported elsewhere [5, 6, 8]. Briefly, anti-malarials identified during the outlet drug audit were classified according to information on drug formulation, active ingredients and strengths. Artemisinin monotherapies were further classified as oral and non-oral, the latter including medicines recommended for the first-line treatment of severe malaria. Anti-malarial market composition was defined as the proportion of outlets of each type, or the proportion of outlets within each domain, among outlets with oral AMT in stock on the day of the survey. Market share, or the relative distribution of the anti-malarials to individual consumers recorded in the drug audit, was standardized to allow meaningful comparisons between anti-malarials with different treatment courses and different formulations. The adult equivalent treatment dose (AETD) was defined as the amount of active ingredient required to treat an adult weighing 60 kg according to WHO treatment guidelines [1]. Provider reports on the amount of the drug sold or distributed during the week preceding the survey were used to calculate volumes according to type of anti-malarial. The volume of each drug was calculated as the number of AETDs that were reported to have been sold/distributed during the week preceding the survey. Measures of volume included all dosage forms to provide a complete assessment of anti-malarial market share.

The price of oral AMT is reported as the median price per 50 mg tablet of oral AMT sold. This is a departure from the standard ACTwatch method of calculating price, which is the median price per one adult equivalent treatment dose (AETD). Price was calculated in this manner to provide a more relevant price of oral AMT, given that most commonly this monotherapy is sold as one or two tablets, rather than a full course treatment of 20 tablets. The type of outlets supplying oral AMT to service delivery points was calculated as the percentage of outlets reportedly named by the provider as the distributor of the oral AMT product. Providers had the option to name more than one supplier/source.

### Protection of human subjects

The 2015 outlet survey protocol received ethical approval from PSI’s Research Ethics Board, headquartered in Washington DC, USA and registered under the Office of Human Research Protections (OHRP FWA00009154, IRB#00006961).

## Results

### Availability

Table 1 shows a detailed breakdown of oral AMT availability at the outlet level across domains. A total of 3859 outlets across all four geographic domains had an anti-malarial in stock on the day of survey. Of these, 988 outlets had oral AMT in stock on the day of the survey, and 217 had oral AMT within the past 3 months.

**Table 1:** Availability of oral artemisinin monotherapy by domain

	Eastern		Central		Western		Coastal		Total	
	N		N		N		N		N	
Number of outlets in sample										
Stocking an anti-malarial	1330		594		1065		870		3859	
	**%**	**95% CI**	**%**	**95% CI**	**%**	**95% CI**	**%**	**95% CI**	**%**	**95% CI**
Proportion of anti-malarial-stocking outlets stocking										
Oral AMT in stock or in the past 3 months	16.4	(12.0, 22.1)	22.4	(18.3, 27.2)	41.5	(36.2, 47.0)	15.5	(12.2, 19.4)	20.3	(17.6, 23.0)
Oral AMT in the past 3 months, not on the day of the survey	1.4	(0.9, 2.1)	3.1	(1.7, 5.9)	4.7	(3.4, 6.6)	4.7	(3.0, 7.5)	2.9	(1.9, 3.9)
Oral AMT on the day of the survey	15.0	(10.8, 20.5)	19.3	(15.5, 23.7)	36.8	(32.1, 41.6)	10.7	(8.2, 14.0)	17.3	(14.8, 19.8)

The national estimate for oral AMT availability on the day of the survey among anti-malarial stocking outlets was 17.3%. Observing domain differences, availability was highest among outlets in the Western border at 36.8% versus the Eastern domain (15.0%), Central domain (19.3%), and Coastal domain (10.7%). The percentage of outlets stocking oral AMT in the past 3 months but not on the day of the survey was 2.9% nationally and was comparable across domains.

**Fig. 1:**
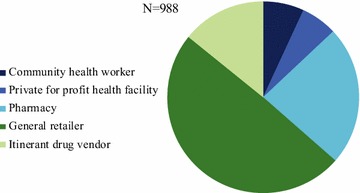
Market compostion  of oral AMT-stocking outlets, by outlet type

**Fig. 2:**
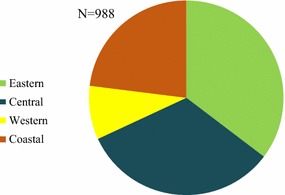
Distribution of oral AMT stocking outlets, by domain

Figures 1 and 2 illustrate the relative distribution of service delivery points for oral AMT stocking outlets, by domain and by outlet type. Figure 1 shows that in terms of absolute number of places where oral AMT medicines were available, most were categorized as private sector outlets. Over 90% of the oral AMT service delivery points comprised of private sector outlets: general retailers (49.4%), pharmacies (23.5%), and itinerant drug vendors (14.2%). The relative distribution of oral AMT-stocking outlets by outlet type was less than 10% for community health workers and private for-profit facilities (6.9 and 6.1%, respectively). Of the 988 oral AMT-stocking outlets nationally, the Eastern area had the largest relative number of oral AMT-stocking outlets nationally at 35.3%, followed by the Central area (32.7%). The relative number of oral AMT-stocking outlets was 23.0% in the Coastal domain and less than 10% in the Western domain (Fig. 2).

**Table 2:** Product catalogue of the types and number of oral AMT products

Country of manufacturer	Manufacturer	Brand name	Strength (mg)	Package size	N
Artemether tablets					68
China	Kunming Pharmaceutical Corporation (KPC)	Artem	40	12	26
	Chongqing Holley Pharmaceutical Co. Ltd.	Artemether	40	12	7
Vietnam	AA medical products	AA-Artemether	50	12	22
–	–	Artemether	50	12	2
–	–	Artemether	–	–	11
Artesunate tablets					968
China	Guilin Pharmaceutical Co. Ltd.		50	12	2
	Jiangxi Xierkangtai Pharmaceutical Co. Ltd.		50	12	1
	Zhangfeng Pharmaceutical Factory		50	12	17
	Zhejiang Holley Nanhu Pharmaceutical Co. Ltd.	Arthesis 50 mg	50	12	12
Myanmar	Myanmar/Tatmadaw Pharmaceutical Factory		50	100	14
Vietnam	AA medical products	AA-Artesunat	50	12	17
	Central Pharmaceutical Factory No. 1	Artesunate	50	12	2
	Mediplantex		50	12	834
	Medopharm		50	12	2
	Mekophar Chemical Products		50	12	1
	Traphaco	Traphasunat 50 mg	50	12	9
–	–	Artesunate	50	12	18
–	–	Artesunate	–	–	39
Total oral AMT					1036

Table 2 shows a breakdown of audited oral AMT products. Of the 1036 oral AMT products audited (Eastern, N = 308; Central, N = 166, Coastal, N = 180, Western, N = 648), tablet formulation was either artemether (68 products) or artesunate (968 products). The most commonly audited oral AMT product was branded as Artesunate^®^, manufactured by Mediplantex in Vietnam (n = 834). Other common products included Artem^®^ by Kunming Pharmaceutical Corporation in China (n = 26) and AA-Artemether^®^ by AA medical products in Vietnam (n = 22). All artesunate tablet products with known product information had a strength of 50 mg (n = 929) whereas artemether tablets with known product information were found in strengths of 40 mg (n = 33) and 50 mg (n = 24). All branded oral AMT products came in a pack size of 12 tablets with the exception of Artesunate^®^ manufactured by Myanmar Pharmaceutical Factory or Tatmadaw Pharmaceutical Factory in Myanmar that were found in tins of 100 tablets (n = 14). There were 50 oral AMT products that were found as loose tablets stored in containers (plastic tins) without product information (brand, manufacturer, and country of manufacturer), represented by two dashes in the table.

**Table 3:** Characteristics of oral AMT supply and demand

	National	
	%	(95% CI)
Proportion of oral AMT products that were reportedly obtained from	N = 1335	
Pharmacy	53.5	(47.3, 59.7)
Fixed-location medicine wholesale outlet	40.0	(33.5, 46.6)
Other (*mobile distributor, village buyer, rural health centres, and ‘floating shops’*)	8.1	(5.3, 10.9)
Proportion of oral AMT products that were reportedly obtained from a supplier within the past 3 months	53.1	(47.1, 59.0)
	**Median**	**[IQR]** ^**(N)**^
Among oral AMT products, median		
Number of oral AMT packages in stock on the day of the survey	1.0	[0.4–2.4]^(1302)^
	**%**	**(95% CI)**
Proportion of oral AMT products* that		
Were reportedly requested by name by consumers	68.2	(63.0, 73.4)

*Oral AMT products in stock on the day of the survey or reportedly in stock in the past three months

The characteristics of oral AMT supply and demand are shown in Table 3. According to provider reporting, most oral AMT products were obtained from a pharmacy (53.5%) or a fixed-location medicine wholesale outlet (40.0%). Only 8.1% of oral AMT products were obtained from some other type of supplier; the most common other types of suppliers cited were mobile distributor, village buyer, rural health centre, or ‘floating shops’ which were reportedly found on boats that traverse local rivers. Over half (53.1%) of oral AMT products had been obtained from a supplier within the past 3 months. The median number of packages in stock on the day of the survey was 1.0 (IQR = 0.4–2.4). Over two-thirds (68.2%) of oral AMT products were reportedly requested by name by consumers.

**Table 4:** Characteristics of oral AMT products: expiry, manufacturer, and price

	%	(95% CI)
Proportion of oral AMT products		
Expiry dates^Ψ^	N = 912	
Expired at the time of the survey	14.7	(9.8, 19.7)
Less than 1 year to expiry	23.6	(18.3, 29.0)
1–2 years to expiry	9.7	(5.7, 13.8)
Greater than 2 years to expiry	60.4	(54.2, 66.6)
Manufacturer	N = 1036	
Mediplantex (Vietnam)	75.0	(68.9, 81.1)
AA medical products (Vietnam)	8.0	(4.7, 11.2)
Kunming Pharmaceutical Corporation (China)	2.6	(1.0, 4.2)
Other	14.4	(10.1, 18.7)
	**Median**	**[IQR]** ^**(N)**^
Among oral AMT products, median		
Retail price per 50 mg tablet sold to patients	$0.16	[0.12–0.18]^(989)^
Retail percentage mark-up	30.1%	[14.9–54.2%]^(817)^
Number of 50 mg tablets typically dispensed to treat malaria in an adult	2	[1.2–7.2]^(928)^

Ψ There were 124 oral AMT products missing expiry date information; these were not included in the denominator

Table 4 displays the breakdown of oral AMT product characteristics. Of the 1036 oral AMT products in stock on the day of the survey, 75.0% was Artesunate^®^, manufactured by Mediplantex in Vietnam. Another 8.0% of oral AMT products were manufactured by AA medical products in Vietnam and were either AA-Artesunat^®^ (n = 17) or AA-Artemether^®^ (n = 22). Kunming Pharmaceutical Corporation in China was responsible for manufacturing 2.6% of oral AMT products (all were Artem^®^) found in Myanmar in 2015.

At the time of the survey, of the 912 oral AMT products with known expiry dates, 14.7% of oral AMT products audited were expired and slightly less than a quarter (23.6%) had less than a year to expiry. Over 60% of oral AMT products found at the time of the survey still had a shelf life of greater than 2 years. When broken down by manufacturer, 72.5% of Artesunate^®^ by Mediplantex had a shelf life of greater than 2 years. Over 85% of products manufactured by AA Medical were expired or due to expire within the year, and almost 85% of oral AMT that had been manufactured in China was expired at the time of the survey. The median number of oral AMT tablets typically dispensed to treat malaria in an adult was two tablets, approximately 10% of an AETD, and had an IQR of 1.2–7.2 tablets. The median price of a 50 mg tablet after the median 30.1% retail percentage mark-up was $0.16. This equated to a treatment price of $0.32 for a typical oral AMT treatment dose.

**Fig. 3:**
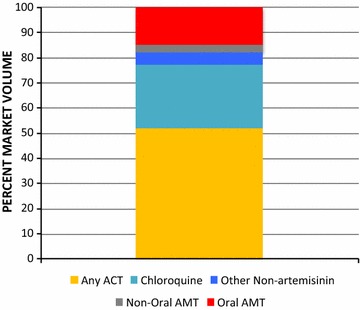
National market share of different classes of anti-malarials

**Fig. 4:**
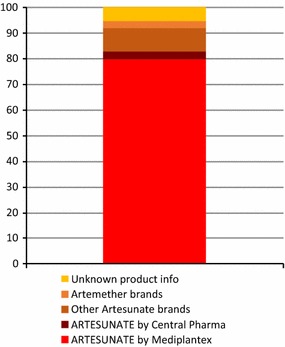
Market share by oral AMT product type

Figure 3 shows the national level anti-malarial market share for each type of anti-malarial distributed: ACT, chloroquine, other non-artemisinin therapies, and non-oral and oral artemisinin monotherapies. Figure 4 illustrates the oral AMT market share for artemether and artesunate and for major brand names. Sale or distribution of oral AMT comprised 14.8% of the anti-malarial market nationally in 2015. Of all oral AMT sold or distributed, 79.9% was labelled Artesunate^®^ and manufactured by Mediplantex in Vietnam. Other products that were sold in low volumes included Artesunate^®^ by Central Pharmaceutical Company in Vietnam, and Artem^®^ by Kunming Pharmaceutical Corporation in China. Around 5% of the oral AMT products had unknown brand and manufacturing information.

## Discussion

This study provided timely, actionable evidence to address persistent sale and distribution of oral AMT in Myanmar. The results of the study illustrate widespread availability and distribution of oral AMT across Myanmar. Strategies to confiscate this medicine from the marketplace, and to scale-up existing efforts to completely remove this monotherapy are urgently needed. Lessons learnt from other countries can be applied to ensure successful strategies, though must be adapted to meet Myanmar’s ethnically diverse and vastly different geographical landscape.

### Where is oral artemisinin monotherapy available?

Findings from the 2015 ACTwatch outlet survey found a high number of oral artemisinin monotherapies, with over 1000 audited products representing over 10% of all anti-malarial medicines audited. The findings illustrate how the private sector remains responsible for the majority of oral AMT distribution in Myanmar. General retailers, pharmacies, and itinerant drug vendors were the outlet types most commonly responsible for stocking oral AMT. This is consistent with 2015 market share observations that show oral AMT market share was markedly higher in the total private sector (19.6%) versus community health workers (3.8%) [9]. Future strategies to address the removal of this medicine must target and engage with the private sector, including pharmacies, general retailers and itinerant drug vendors.

At the national level, one in five anti-malarial-stocking outlets had oral AMT in stock. Close to half of anti-malarial-stocking private sector outlets in the Western domain were stocking oral AMT, pointing to widespread availability in areas next to the Indian border. Although the oral-AMT availability was highest in the Western domain, the majority of oral AMT-stocking outlets were located in the more populous Eastern and Central areas of the country. Oral AMT availability across the country is of grave concern given Myanmar is considered an important probable origin and route of spread of multi-drug resistance to other parts of the world.

The results are somewhat surprising in Eastern Myanmar, given that several strategies have been implemented in this area since 2012 to contain the spread of artemisinin drug resistance [10]. One of the key strategies has been a project to remove oral AMT from the private sector and replace it with quality-assured ACT through the AMTR project. In 2012 the main distributor of oral AMT, AA Pharmaceuticals, was identified and engaged in an agreement to replace sales of oral AMT with first-line, subsidized ACT. Additional strategies included behaviour change communication (BCC) to increase demand for ACT, coupled with intensive pharmaceutical detailing operations, targeting general retailers, pharmacies and itinerant drug vendors through product promoters to facilitate ACT uptake in the supply chain. These efforts were coupled by the significant role of the Myanmar FDA, which has played an important role in trying to enforce the ban on oral AMT registration and importation despite limited resources and challenges in gaining access to border control areas. This included high-level advocacy with the military-operated Tatmadaw Pharmaceutical to halt the manufacturing of oral AMT in the country in late 2015. It is expected that the last batch of oral AMT manufactured by Tatmadaw was produced in late 2015 as an agreement was drawn up between the Ministry of Health (MoH) and the Ministry of Defense. Indeed, this locally manufactured product was rarely audited in the survey.

Despite these efforts by the government and international NGOs, market trend data from the Eastern part of Myanmar showed that while availability of oral AMT has declined over the years, between 2014 and the current study round, an increase in availability was observed among general retailers, pharmacies and itinerant drug vendors from 10.3% in 2014 to 27.2%. That said, availability of oral AMT was lowest in the Eastern domain as compared to other domains, pointing to the success of the many strategies. It is postulated that in 2015 there may have been a final push by distributors to get rid of any last remaining stock of oral AMT, before it expired. Perhaps more likely, this could reflect a 3–4 month stock-out of ACT medicines during the peak malaria transmission season in 2015. Frequent changes in key leadership positions within the Ministry of Health resulted in an unexpected delay in obtaining approval to import ACT medicines into the country  for the AMTR project, indicating the importance of constant supply of ACT to ensure universal coverage and reduce the distribution of unwanted medicines.

Other higher level, multi-pronged strategies may be considered that speak to enforcement and regulation of the ban. Strategies have recently been implemented in Cambodia resulting in the successful removal of oral AMT from the private sector [11]. These included increasing private sector access to subsidized first-line ACT. To enforce the ban on oral AMT instituted in 2008, strategies have included the recruitment of officials known as the ‘Justice Police’, as well as training of over 400 police to identify oral AMT at service delivery points and actively enforce the ban by removing products and sanctioning outlets. The government also used mystery clients to further identify outlets selling any oral AMT, and instituted a system of licensing drug outlets and using the Justice Police to regularly check for and confiscate fake, substandard or banned drugs. In addition, an international collaboration led by INTERPOL succeeded in interrupting one trade route of a major Chinese producer of fake artemisinin that had flooded the market in Cambodia [12]. These efforts were supported by a communication campaign to educate private providers and consumers about the dangers of oral AMT. Similar success stories have also been observed in the Lao Peoples Democratic Republic, where the availability of oral AMT significantly decreased from 22.9% in southern Laos in 2003 to 4.8% in 2012 [13]. This was attributed to several strategies to improve case management in the private sector, but also reflected changes to treatment policy, trading agreements with manufacturers of oral AMT, and amendments to the law on Drugs and Medical Products to ensure adequate registration, distribution, and supply of all medicines.

Such strategies may be applicable in Myanmar to ensure effective and immediate removal of oral AMT from the marketplace. To-date, there has been no official communication on oral AMT in Myanmar, including a lack of communication on banned products and the dangers of continuing use of such products. Communication campaigns could be an important first step to tackle the widespread availability of oral AMT. However, these interventions must be couched with the reality that Myanmar has over 100 ethnic groups, with many different languages, which can pose a challenge for clear and effective communication. Future strategies must also consider that many areas of Myanmar are under conflict and controlled by ethnic armed groups, for which the central government and local and international NGOs have variable access. The political and geographical diversity must be considered and overcome to ensure effective implementation and successful behaviour change strategies.

Promisingly, the Myanmar FDA underwent a major restructuring of their department in 2014 and the Government of Myanmar has allocated considerable resources to improve the infrastructure, including an expansion of human resources. In addition to the Central level, the FDA now has officers at the State and Division level, allowing for greater national influence. This presents an opportunity for implementers to work closely with the FDA as a means to strengthen and regulate the sale of oral AMT and educate wholesale providers about the dangers of oral AMT.

### What types of products are found on the shelf and being distributed?

All oral AMT products found in Myanmar were manufactured in either Vietnam, China, or locally in Myanmar, with the vast majority having been manufactured in Vietnam. Three-quarters of the available products were Artesunate^®^, manufactured by Mediplantex in Vietnam. This was also the most commonly distributed product by far, capturing over 80% of the market share. These products were stamped with ‘GMP-WHO’ (Good Manufacturing Practices-World Health Organization), misleadingly indicating to both patients and providers that these drugs are recommended by the WHO based on the implicit level of quality conferred by the manufacturer’s GMP status. This is of further concern given that ‘GMP-WHO’ is a fake label and does not exist as a quality standard. Additionally, the instructions were only in Chinese and English—with no Myanmar translation—and also included vague contraindications, such as ‘pregnant women should use the drug carefully’.

With many products having a shelf life of greater than 2 years at the time of the survey, and some products with an import registration number, this confirms that there has been both recent manufacturing of the product as well as recent importation. Indeed, direct communication with the manufacturer confirmed that oral AMT was still in production in late 2016, with two strengths (50 and 100 mg), with a minimum order of 20,000 tablets [14].

The findings from the product audit speak to three important conclusions regarding importation and future strategies to remove the medicine. First, the recent import of this medicine confirms that there are significant gaps or loopholes in the regulatory policy surrounding oral AMT, either that the ban on import is not being enforced, or simply that the import of oral AMT products with a registration license is still legal in Myanmar. Myanmar can potentially expect further import of this medicine until the manufacturer decides to stop producing it. Second, as no other single brand had more than three percent of the market share, another key conclusion from this study is that currently a single product and manufacturer dominates the market. This provides an opportunity, not dissimilar from that of the situation with a key supplier identified in the Eastern part of the country several years ago [10], to target the top of the supply chain and halt further large-scale distribution of this product. Finally, while oral AMT manufacturers were limited to three countries, the wider range of brand names and one-off products, seems to suggest that oral AMT is not only being imported under official supply chains (e.g. products with registration numbers) but through unofficial channels across the border. Increased regulation of borders or licensing of medicines will be necessary to ensure that removal of oral AMT is sustained, which could be addressed nationally by explicit communication from the FDA to wholesalers regarding the dangers of oral AMT. This should also be addressed by WHO as part of their policy to halt the manufacturing of oral AMT. On a yearly basis, WHO routinely reaches out to manufacturers of oral AMT and asks them to sign agreements to stop production of this medicine [4].

### What are the provider practices regarding the re-supply and sale of oral artemisinin monotherapy?

Oral AMT was reportedly obtained by providers from a limited number of supplier types. Pharmacies and fixed-location medicine wholesale outlets were the supplier for 93.0% of available products. Most outlets had a small number of oral AMT packages in stock and reported fairly frequent re-stocking with small volume purchases. The limited number of supplier types and minimal amount of oral AMT products in stock at the lowest level suggests that the oral AMT supply chain could be rapidly disrupted. This would additionally benefit from several strategies mentioned previously to ensure tightened regulation of the policy.

As providers typically had only one or two packages in stock, it is perhaps not a surprise that oral AMT was most often distributed in doses of approximately two tablets, making it feasible for a provider to treat up to six patients with a 12-tablet package. Sub-optimal dosing of oral AMT medicines is particularly rampant throughout the private sector. This is not only unsafe for the patient, but also dangerous over time as the selection pressure on parasites to develop resistance to artemisinin is quite high [15]. Evidence of artemisinin-resistance is now prevalent throughout much of Myanmar where malaria is endemic and the spread of molecular markers across Myanmar has now been detected on the border with India [16]. Continued distribution of oral AMT at such a low dosage will further exacerbate the prevalence of artemisinin resistant malaria parasites in Myanmar and increase the likelihood of spread of artemisinin resistance into China, India, and westward [17].

More than two-thirds of oral AMT products were reportedly requested by name by patients, suggesting that consumer demand may also play a role. A recent study that investigated factors associated with outlet providers who stock oral AMT in Myanmar, found that the odds of stocking oral AMT was almost four times higher among providers who report that customers ask for this by name compared to providers that report costumers do not demand the medicine by name [18]. Evidence from other countries also supports this, where administration of the first-line treatment seems to depend on what patients demand [19]. This points to the need for strategies that not only focus on provider behaviour change, but also address patient preferences and awareness of what medicine to ask for. As such, targeting provider knowledge, preferences and behaviour alone may not be sufficient to drive uptake of the first-line treatment [20]. To help remove oral AMT from the market, there is likely a need for supporting interventions to drive consumer awareness and demand for first-line treatment [21].

The policy implications from this paper are clear. The production of oral AMT by Vietnamese manufacturers must end. The continued manufacturing and distribution not only threatens efforts by WHO efforts to combat artemisinin resistance, but also threatens malaria control efforts in Africa. It is both discouraging and disheartening that while oral AMT consumption is banned in Vietnam, manufacturing and exportation is however permitted [WWARN, pers. comm.]. These disappointing actions should act as a rallying call to the global community, especially given the financial investment and international and national commitment to preserve the efficacy of artemisinins and ultimately save lives. Continued production of oral AMT should be raised before the World Health Assembly as a means to gather further momentum by the international community to stop production of this dangerous anti-malarial.

Finally, as the sale of oral AMT is typically just two tablets, the price of a typical dose has implications for pricing quality-assured recommended alternatives. While a private sector subsidy has reduced the price of quality-assured ACT in Myanmar, a typical dose of oral AMT remains a less expensive option for a patient and provides a greater profit margin for the provider than first-line ACT for treatment. The wholesale to retail markup of oral AMT was much higher than that of an ACT in the private sector: 30.2 versus 20.0% for ACT in the context of an ACT subsidy programme [7]. Coupled with the knowledge that oral AMT shelf life is also double that of ACT (approximately 4 versus 2 years, respectively) [10], evidence suggests a number of financial incentives for providers to stock and sell this monotherapy over an ACT. Furthermore, the median treatment price for a typical oral AMT treatment dose of two tablets was $0.32. This is comparable, and is in fact more than 10% less expensive than the median price of $0.36 for ACT in Myanmar, suggesting that oral AMT may be more affordable to patients. Consumer price and potential profit for providers may be factors influencing demand for oral AMT in Myanmar, as has been identified in other settings introducing ACT in competition with cheaper anti-malarial alternatives [8, 22, 23]. Increased provider and consumer knowledge on the differing treatment guidelines between *Plasmodium falciparum* and *Plasmodium vivax* malaria, could create market demand for appropriate treatment for each malaria species according to national guidelines.

## Limitations

The 2015 ACTwatch Outlet Survey in Myanmar was not designed to capture the entire anti-malarial market or to map the entire supply chain for oral AMT and for all anti-malarial medicines. The study was limited to the private sector and community health workers—government facilities were not visited. However, it is likely that oral AMT availability and distribution are relatively low in government health facilities. This study used the adult equivalent treatment dose (AETD) for the unit of analysis in calculating relative market share for each type of anti-malarial medicine. However, oral AMT is most typically sold in a treatment dose of two tablets instead of the full AETD of about 20 tablets. As such, the proportion of patients that are treated with oral AMT relative to other types of anti-malarials is likely much higher than the market share estimated using AETDs distributed. Given the widespread availability and distribution of oral AMT, further study is needed to understand the supply chain at various levels. This is particularly true at higher levels in order to understand where and under what guise oral AMT is imported. Additional surveys in key areas of China or along the India border would be particularly beneficial. The study also did not assess consumer demand or behaviour directly. Further studies that engage the patient, especially in a qualitative manner, are also necessary to inform effective strategies to address consumer knowledge and behaviour.

## Conclusion

Given the high availability and distribution of oral AMT, it is possible that Myanmar has become the last remaining viable market for any oral AMT in the region that is not expired or is still being manufactured. This could be due to loopholes in Myanmar’s regulatory policy and/or stringent regulations in the manufacturing country that do not permit local sale of oral AMT. Other factors that contribute to the uptake and ongoing distribution of this medicine speak to the long shelf-life of the product, provider dispensing practices, price and consumer demand. Urgent action is required to stop the manufacturing, sale and distribution of this medicine to protect the efficacy of ACT and curb the spread of multi-drug resistant parasites. A multi-pronged approach is needed to address the problem. Targeting suppliers at the top of the supply chain, improving and expanding upon BCC activities for both the provider and consumer, and demand for appropriate medicines are all crucial. International advocacy is also urgently needed to stop the manufacturing and exportation of oral AMT in Vietnam and China, and advocacy measures by WHO are needed to encourage manufacturers to halt production. National policy changes that ban the full import, distribution, and sale of oral AMT and ultimately implementation of a programme for removal of oral AMT from the market would create a far more conducive environment for the support of these activities.

## Additional files

**Additional file 1.** Description of outlet types and classification

## Abbreviations

AETD: adult equivalent treatment dose
AMT: artemisinin monotherapy
ACT: artemisinin-based combination therapy
AMTR: artemisinin monotherapy replacement
BCC: behaviour change communication
FDA: Myanmar Food and Drug Administration
GMS: Greater Mekong Sub-region
MoH: Ministry of Health
PPS: probability proportional to population size
RDT: Rapid diagnostic test
WHO: World Health Organization
