# Supplementary material for: Insights into the availability and distribution of oral artemisinin monotherapy in Myanmar: evidence from a nationally representative outlet survey
Source: Malar J. 2017 Apr 25;16:170. doi: 10.1186/s12936-017-1793-0 (PMC5404336; doi:10.1186/s12936-017-1793-0)
Supplement: Supplementary file 1 — Additional file 1. Description of outlet types and classification [file 12936_2017_1793_MOESM1_ESM.docx]

Additional file 1: Description of outlet types and classification

| Community health worker | Community-based health workers provide patient services and typically are linked with the government or NGOs, or other private health facilities and medical supply agents. |
| --- | --- |
|  |  |
| Private for-profit health facilities | Private general practitioners are providing patient services within privately owned facilities that are licensed by the Ministry of Health. These practitioners may have formal or informal ties with government health facilities including serving on staff at government facilities and/or accessing government or non-government not-for-profit medicine supplies. |
| Pharmacies | Pharmacies are licensed by the Ministry of Health and are authorized to sell all classes of medicines including prescription-only medicines. |
| General retailers | General retailers are grocery stores and village shops that sell fast-moving consumer goods, food and provisions. Although retailers may have over-the-counter medicines including anti-malarials available, national authorities do not regulate the sale of medicines by retailers. |
| Itinerant drug vendors | Mobile providers selling medicines and other goods. They are not registered with any national regulatory authority. |
|  |  |
|  |  |
|  |  |
